# Understanding acute burn injury as a chronic disease

**DOI:** 10.1186/s41038-019-0163-2

**Published:** 2019-09-16

**Authors:** Lucy W. Barrett, Vanessa S. Fear, Jason C. Waithman, Fiona M. Wood, Mark W. Fear

**Affiliations:** 1 Telethon Kids Institute, University of Western Australia, Northern Entrance, Perth Children’s Hospital, 15 Hospital Ave, Nedlands, WA 6009 Australia; 2 Institute for Respiratory Health, Ground Floor, E Block Sir Charles Gairdner Hospital, Hospital Avenue, Nedlands, WA 6009 Australia; 3 Fiona Wood Foundation, Fiona Stanley Hospital, MNH (B) Main Hospital, CD 15, Level 4, Burns Unit, 102-118 Murdoch Drive, Murdoch, WA 6150 Australia; 4 Burns Service of Western Australia, WA Department of Health, Nedlands, WA 6009 Australia; 5 Burn injury research unit, School of Biomedical Sciences, University of Western Australia, Crawley, WA 6009 Australia

**Keywords:** Burns, Immune system, Endocrine system, Homeostasis, Patient care, Chronic disease

## Abstract

While treatment for burn injury has improved significantly over the past few decades, reducing mortality and improving patient outcomes, recent evidence has revealed that burn injury is associated with a number of secondary pathologies, many of which arise long after the initial injury has healed. Population studies have linked burn injury with increased risk of cancer, cardiovascular disease, nervous system disorders, diabetes, musculoskeletal disorders, gastrointestinal disease, infections, anxiety and depression. The wide range of secondary pathologies indicates that burn can cause sustained disruption of homeostasis, presenting new challenges for post-burn care. Understanding burn injury as a chronic disease will improve patient care, providing evidence for better long-term support and monitoring of patients. Through focused research into the mechanisms underpinning long-term dysfunction, a better understanding of burn injury pathology may help with the development of preventative treatments to improve long-term health outcomes. The review will outline evidence of long-term health effects, possible mechanisms linking burn injury to long-term health and current research into burns as a chronic disease.

## Background

Burn injury is a major public health issue, with an estimated 11 million incidences globally per year resulting in more than 300,000 deaths [1]. Burns are complex traumatic injuries, and much of the focus of research and clinical treatment has been on the acute trauma, appropriate surgical intervention and survival with reduced scarring. However, it is increasingly being acknowledged that burn injury can result in sustained and severe physiological and psychological problems. Some of these long-term effects have been well documented in the clinic, stemming from the prolonged healing period and the resulting physical scars. Other long-term health effects have been less well described. Recently, there has been increasing evidence of long-term health effects of a burn injury. Notably, the long-term effects have been observed after both severe and non-severe burns (< 20% total body surface area (TBSA)). This is significant, as the vast majority of burn patients, particularly in developed countries, suffer non-severe injuries. The review will outline evidence of long-term health effects, possible mechanisms linking burn injury to long-term health and current research into burns as a chronic disease.

Our initial literature search involved searching PubMed for articles containing the words “burn” AND “long-term”. This search returned 1274 references, 170 of which were identified as relevant to the topic of long-term health impacts of burn injury. Of these 170 references, 68 were about the long-term effects on mental health (the most well-known impact and therefore not a major focus of this review), 41 were discussing the long-term impacts of specific treatment regimens or specific types of burn and 30 were referring to what we consider to be acute stage (< 1 year post-burn). The remaining 31 references were all used in this review. The relatively small number of relevant publications returned by this search is indicative of the lack of research in this area, mainly due to the fact that many of the secondary pathologies discussed in this review were only linked to burn recently by long-term population studies. However, the data from these recently published studies will undoubtedly guide future research and lead to a better understanding of the overall impact of burn injury.

## Review

### Long-term pathophysiology of burn injury

#### Metabolic changes, scarring and mental health disorders

Compared to other traumatic injuries, burn patients face a prolonged healing process and are often left with physical and mental scars. Hypermetabolism is a well-characterised acute impact of burn [2]; however, recent evidence has shown that these changes persist in some manner years after the initial injury (reviewed in [3]). A study of 977 paediatric patients with severe burns analysed a variety of clinical markers and found that patients were still in a hypermetabolic state 3 years post-injury [4]. The persistence of the hypermetabolic state results in sustained loss of muscle mass and bone density [5, 6]. An increase in muscle protein synthesis occurs in this hypermetabolic state, with a higher rate of protein degradation resulting in chronic amino acid loss that is sustained up to 1 year post-burn injury [7]. The respiratory capacity of muscle mitochondria also remains significantly reduced in burn patients 1 year post-injury [8], and muscle strength in patients with severe burns remains weaker at 1–5 years post-burn follow-up [9]. Loss of bone density as a result of inflammatory bone resorption and osteoblast apoptosis in paediatric patients with severe burns also persists long after the initial healing process [10].

While mortality rates for burn patients have significantly improved, hypertrophic scarring is a major long-term concern for survivors, especially for paediatric patients and patients suffering severe burns. Burn healing results in the deposition of excessive and disorganised extracellular matrix, reducing the pliability of scars. In hypertrophic scar, myofibroblasts persisting in the wound post-healing leads to continued contraction [11]. Treatments for scar include compression garments, massage, laser therapy, steroids and surgery [12], but there is a continued need for targeted therapies to reduce scar burden. Surgery may be required for hypertrophic scars that do not respond to other treatments, as depending on the location of the injury, scars can significantly impact movement and joint function.

Because of the context and severity of burn injuries, patients often suffer mental health problems during and long after the acute healing phase. Mental health disorders including post-traumatic stress disorder (PTSD) have been reported in burn patients more than a year after injury [13], and in one study of 90 burn patients 1–4 years postburn injury, 10% of patients suffered from major depression, 10% from anxiety and 7% from PTSD [14, 15]. Patients with severe burns also frequently suffer from chronic persistent pain, which can have a significant impact on patient well-being in daily life. In a survey of 358 patients with severe burns, 52% of respondents reported suffering ongoing burn-related pain, despite their injuries occurring an average of 11 years prior [16]. The associated physical scars that remain after the burn has healed also contribute significantly to the pain and mental distress experienced by these patients [17].

### Population studies identify long-term health impacts of burn injury

**Fig. 1:**
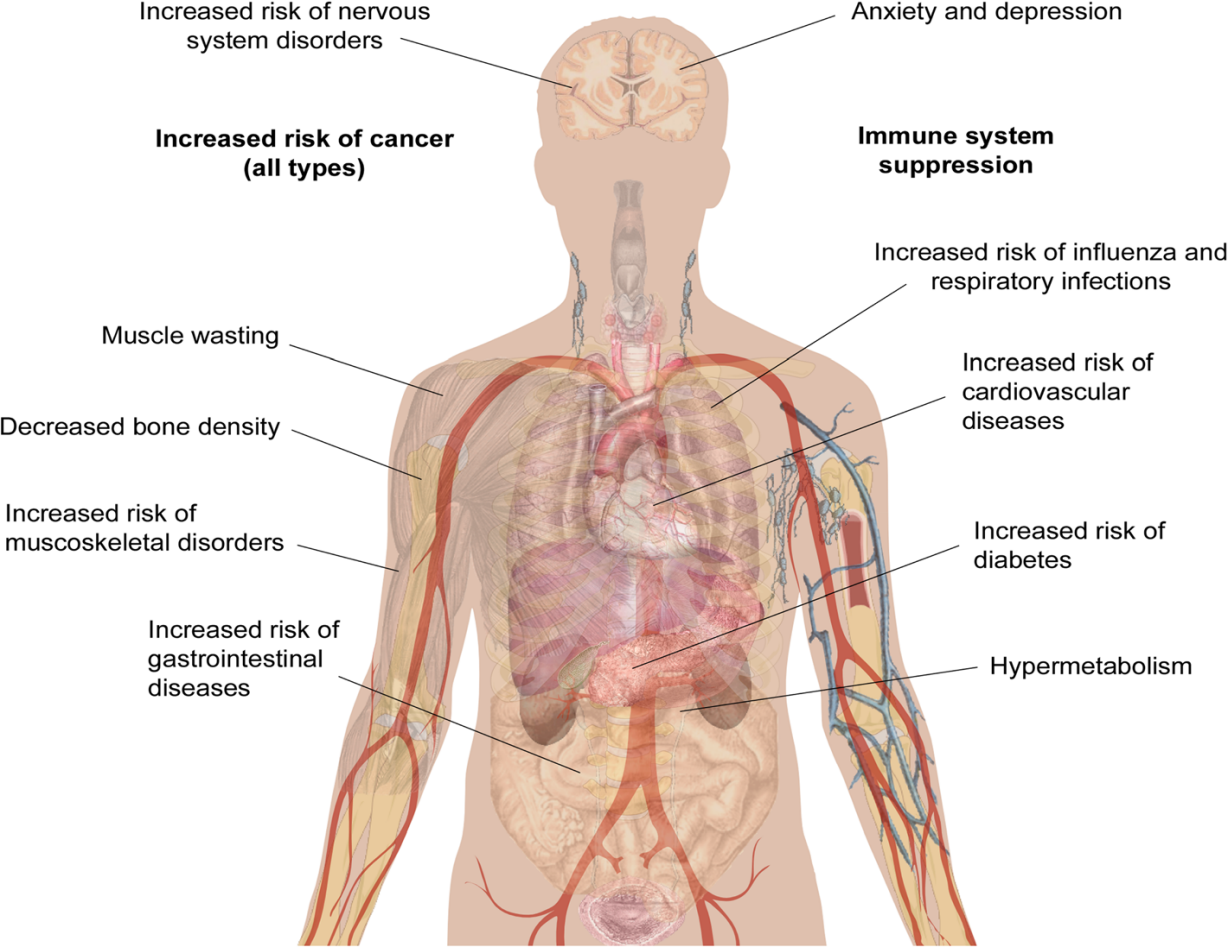
Long-term pathological effects of burn injury. Burn injury is associated with an increased risk of numerous secondary pathologies. The human body schematic is a copyright free image obtained from google images

While clinical observations of hypermetabolism and the effects of burn injury on mental health and chronic pain have been reported for a number of years, other long-term impacts of burn injury have only recently been uncovered. The Western Australian (WA) Population-based Burn Injury Project is the most comprehensive long-term study of burn injury to date. This project undertaken by researchers from the Fiona Wood Foundation used linked hospital morbidity and death data from Western Australia from all patients hospitalised for a first burn injury from 1980 to 2012 (*n* = 30,997) and a randomly selected, frequency matched uninjured comparison cohort (*n* = 127,000). The burn injuries included minor (49% of patients) and severe burns (4%) (the severity of the remaining 47% were unspecified), with a range of depths. The scope of this data has allowed the investigation of the long-term impact of burn from many different angles. The major findings of these studies are summarised below and in Fig. 1, and the potential cause(s) of these correlations will be discussed in more detail later.

#### Increased mortality

One of the first findings from the WA studies was that burn injury that requires hospitalisation results in higher long-term mortality rates for both children and adults. Paediatric burn patients had a 1.6 times (1.6×) higher age-adjusted mortality rate when compared to uninjured children over the 33-year study period, and this risk was increased in patients with severe burns compared to minor burns [18]. This increase in mortality was also seen in adolescents, young and middle-aged adults (aged 15–44 at the time of injury), who had a 1.8× higher mortality rate than observed in the uninjured cohort [19], and in older adults (45+), who had a 1.4× higher mortality rate [20]. Middle-aged and older adults who died during the follow-up period from the burn cohort were also statistically significantly younger than those in the uninjured cohort (43 vs 47 [19] and 76 vs 82 [20]). In support of these findings, another recent population study followed 1965 burn survivors and 8671 matched controls (mean age 44 years) for a median of 5 years. They found that the 5-year mortality was significantly increased among burn survivors, from 4% in controls to 11% in burn survivors [21].

Interestingly, comparing the effects of minor and severe burns in adults, minor burns were associated with a larger increase in mortality. This observation is supported by another hospital study that followed 365 critically ill adult burn patients who survived to hospital discharge found that patients with less severe burns had increased 5-year mortality compared to survivors with major burns [22]. A reason for this may be that individuals who survive major burns are strong physiologically, which provides a survival advantage post-hospital discharge. Another significant finding of these mortality studies is that in the adolescent, young and middle-aged adult cohort, females were found to have a higher increase in mortality compared to males [19]. The causes of death are varied and burn patients appear to be more at risk from deaths of all causes, including accidental and violent deaths [23].

#### Increased risk of disease

The population study revealed that burn patients frequently return to hospital for other conditions, indicating burn injury is associated with an increased risk of disease. These links are discussed below.

#### Cancer—all types

Duke et al. analyzed a sub-cohort of burn patients who were admitted to hospital between 1983 and 1987 (chosen as this group has the optimum follow-up time), which showed there was a 1.39× increase in cancer incidence in females compared to the matched uninjured cohort [24]. In this study, TBSA of the burn but not burn depth was associated with increased risk, with patients with severe burns found to have a 1.81× increased risk of cancer of all types. To strengthen this data, a second cohort of burn patients from Scotland was analysed. This cohort consisted of more than 38,000 patients admitted to hospital and followed up during the period from 1983 to 2008. This study showed a modest but significant increase in overall cancer risk for both genders and increase in cancer incidence in females, confirming the results from the WA study [25]. In this second paper, the types of cancer were also considered. Burn patients across all cohorts, genders and age groups had statistically significant increases in cancer of the buccal cavity, larynx, liver, respiratory tract and oesophagus. In addition, female burn survivors had higher incidences of breast and genital cancer.

#### Infectious disease

Burn injury increases susceptibility to infectious diseases, with higher rates of hospital admissions for infectious diseases found in both severe and minor burns, and the burn cohort was found to have a mortality rate 1.75× higher than the uninjured cohort [26]. Burn patients of all ages were found to have higher admission rates for influenza and viral pneumonia, bacterial pneumonia and other respiratory infections [27]. For these studies, patients with evidence of smoke inhalation of injury to the respiratory tract were removed. Admission rates for respiratory diseases were highest during the first 5 years post-burn; however, they remained elevated compared to the uninjured cohort for the duration of the 33-year study period.

#### Gastrointestinal disease

Boyd et al. and Stevenson et al. showed that both children and adults who experience a burn injury hospitalisation are at increased overall risk of developing gastrointestinal disease [28, 29], which includes diseases of the oesophagus, stomach, duodenum and intestines, noninfective enteritis and colitis, and disorders of the gallbladder, biliary tract and pancreas. The paediatric burn cohort were found to have higher admission rates and spent longer in hospital than the uninjured cohort [28]. These data were similar in adults, who had more admissions and spent longer in hospital than the matched uninjured cohort [29]. This risk in adults was shown to decrease over time; however, rates of hospital admission did remain above the control group for the duration of the study period.

#### Negative impacts on the cardiovascular system

Paediatric burn patients had a higher rate of hospital admissions and days spent in hospital for circulatory diseases compared to the uninjured cohort [30]. Gender-specific analysis revealed this effect is more prominent in boys, with admissions remaining higher more than 20 years after the initial burn injury. A recent study in adolescent survivors of severe burns obtained during childhood found that burn injury is associated with myocardial fibrosis and reduced exercise tolerance [31]; however, more research is needed into non-severe burns and the mechanisms behind this increased risk in paediatric patients.

The increased risk of circulatory diseases was also seen in the adult cohort, with 1.46× more admissions and 2.9× more days spent in hospital [32]. More specifically, adult burn patients had a higher risk of ischaemic heart disease, heart failure and cerebrovascular disease, demonstrating burn injury has long-lasting systemic effects that impact on the heart and circulation [32]. These effects were also maintained in the sub-cohort of adult patients with non-severe burn injury [33].

#### Diabetes

Duke et al. found that the burn cohort had 2.21× more admissions for diabetes mellitus compared to the uninjured cohort. This increase was comparable amongst both genders and in both paediatric and adult patient cohorts and remained elevated for 5 years post-burn, after which there was no significant difference [34].

#### Musculoskeletal diseases

As discussed earlier, burn injury induces negative and sustained impacts on muscle and bone health. Randall et al. demonstrated that burn patients had nearly twice the hospital admission rate for musculoskeletal conditions compared to the uninjured cohort and spent longer in hospital, which included arthropathies, dorsopathies, osteopathies and soft tissue disorders [35]. Rates of fractures were also higher in the burn group, and this was significantly higher in females compared to males [36]. The admission rates for all musculoskeletal disorders remained high for the duration of the study and was elevated in all ages groups [37], demonstrating that both minor and severe burn injuries can affect muscle and bone integrity for at least 20 years post-injury. Holavanahalli et al. that used a self-report measure to investigate musculoskeletal impacts, patients who had sustained burn injuries an average of 17 years earlier reported joint pain and stiffness, problems walking and running and weak arms and hands [38]. The long-term impact of burn on musculoskeletal health has also been recently reviewed in depth [39].

#### Negative long-term impacts on the nervous system

Burn patients of all ages and genders included in the WA study were found to be at risk of nervous system conditions post-burn, with the burn cohort presenting at hospital more frequently than the uninjured cohort and spending 3.25 times the number of days in hospital [40]. Conditions with increased prevalence in burn patients include episodic and paroxysmal disorders such as epilepsy and migraine and nerve, nerve root and plexus disorders [41]. Hospital admissions for these conditions were significantly elevated during the first 5 years post-burn and were found to be sustained in paediatric patients for an extended period of 15 years post-burn.

#### Summary of population studies

The data from the WA population study revealed that burn injury has a wide range of significant long-lasting negative impacts on the overall health of patients and that these effects can also occur after a non-severe burn. This is an important finding and demonstrates the need for a greater understanding of the cellular and molecular effects of burn. The current knowledge regarding the effects of burn on long-term cellular function is discussed in detail in the next section.

### Understanding the long-term impact on endocrine and immune system dysfunction in burn survivors

Burn injury has significant impacts on the endocrine and immune systems, and it is becoming evident that many of these changes are sustained long-term. To date, most long-term studies into these disruptions in burn patients have been done in severely burned paediatric patients. However, the results from the hospital data indicate that patients of all ages with non-severe burns also suffer from these dysfunctions [25, 27]. Hormones are known to influence the immune system, and emerging evidence suggests that the numerous secondary pathologies associated with burn injury are the result of synergistic dysfunctions in these systems, with sustained changes in endocrine homeostasis contributing to long-term immune suppression that is characteristic of burn.

#### Endocrine changes

Following burn there is a rapid release of inflammatory cytokines, catecholamines and cortisol, initiating the hypermetabolic response and catabolic state. A recent study of severely burned children found that levels of urinary norepinephrine and cortisol remained significantly elevated 3 years post-burn [4]. These are stress hormones which inhibit lymphocyte proliferation as well as the activity of CD8+ T cells, natural killer (NK) cells and activated macrophages [42]. They also activate mast cells, leading to degranulation and the release of histamine, which stimulates the production of T helper type 2 (Th2) cytokine interleukin (IL)-10 and causes further vasodilation. Activation of the stress system suppresses the T helper type 1 (Th1) immune response (cellular immunity, generally pro-inflammatory) and favours a Th2 response (humoral immunity, generally anti-inflammatory). A healthy balance of Th1/Th2 responses is a hallmark of a normally functioning immune system and burn clearly disrupts this balance. Although the release of stress hormones is a normal response to trauma, a sustained increase in their expression as seen after burn can have detrimental effects and contribute to long-term immune suppression [43].

Other hormonal changes that occurred after burn in the paediatric study included a significant decrease of serum osteocalcin, parathyroid, insulin growth factor, insulin-like growth factor binding protein-3 and human growth hormone (GH) which were sustained at the 3-year time point and an increase in serum progesterone up to 2 years post-burn, indicative of long-term hormonal imbalance in these patients [4]. The more severe the burn, the greater the dysfunction; one study showed that children with burns > 80% TBSA had higher resting energy expenditure and urinary cortisol levels than patients with smaller burns [44]. Progesterone, which was shown to be increased in patients long after the initial healing process, exerts an immunosuppressive effect, reducing the activity of macrophages and NK cells and promoting a type 2 (Th2) immune response [45]. The Th2 shift may also be driven by the increase in catecholamines, which have been shown to inhibit Th1 and stimulate Th2 cytokine secretion [42]. GH, which is decreased after burn, also modulates the Th1/Th2 responses, with a mouse study looking at the effect of administering GH to burned mice showing that GH increases the production of Th1 cytokines interferon (IFN)-y and IL-2 [46]. It is evident that burn injury disrupts endocrine homeostasis and that this has long-term consequences for immune function.

#### Immune system changes

Compared to non-burn trauma, burn injury triggers a greater and more sustained inflammatory response [47]. Following an initial pro-inflammatory Th1 response where the release of cytokines such as tumor necrosis factor (TNF)-α and IL-6 activates the stress system [46], there is a rapid and sustained increase in IL-10 levels [42]. IL-10 is a Th2 cytokine that induces T regulatory cells and suppresses Th1 responses, leading to a deficient response to infection as a result of reduced cytotoxic T cell activity [48, 49]. IL-10 has also been shown to stimulate the activation of mast cells, promote humoral immunity by differentiating B cells and inhibit macrophage activation and T cell proliferation [42]. In addition to IL-10, a more recent study showed that other Th2 cytokines such as granulocyte-macrophage colony-stimulating factor (GM-CSF), TNF-α, IL-2 and IL-17 also remain elevated up to 3 years post-burn [4]. During the early immune response, there is also increased T regulatory cell activity [50, 51], which is generally indicative of a suppressive immune phenotype.

While immune dysfunction has been recognised in the literature as a consequence of burn injury for more than 2 decades, the persistence of this dysfunction has only recently been investigated. A study investigating the effect of burn injury on immune function analysed cytokine release and immune cell populations in mouse models of burn and excision injuries at different time points [52]. Levels of inflammatory cytokines were measured in the serum of control, burn and excision groups taken on day 1, 3, 7 and 84 postburn injury, and whole blood was taken for analysis of immune cell populations. Comparison between the injury models confirmed that the response to a burn injury as opposed to an excision wound of the same size and depth is significantly different in both the innate and adaptive immune responses. In the acute phase response, the timing and profile of inflammatory cytokine production is significantly different between the two injury models. Increases in monocyte chemoattractant protein 1 (MCP1), MIP1α and MIP1β after burn injury lead to an increased number of monocytes at day 3 post-burn, demonstrating there are changes in immune cell populations early on. Changes in dendritic cell populations at day 28 are indicative of a reduced ability to prime T cells. At the long-term time point (day 84 postburn injury), burn-injured animals sustained a significant increase in IL-10 and decreased total numbers of white cells and lymphocytes in comparison to both control and excision wounded animals [52].

#### Studies in viral infection

Results from the population study highlighted a link between severe and non-severe burn injury and the subsequent development of respiratory infections. This included influenza and bacterial and viral pneumonia. To investigate this link, Fear et al. conducted a study in pre-clinical mouse models to examine the susceptibility to viral infection following a non-severe burn injury [27]. Mice exposed to the influenza virus 4 weeks postburn injury were shown to have increased viral titre in the bronchoalveolar lavage fluid and lung tissue. Analysis of the immune cell subsets showed that the CD8+ T cell proliferative response was diminished, and there were increased numbers of NK and natural killer T cells in the draining lymph nodes, indicating immune cell dysfunction [27]. In another recent murine study, it was found that burned mice were more susceptible to repeated infections which resulted in diminished innate immune cell function and increased anti-inflammatory environment [53].

#### Disruption of homeostasis and heart disease

Aside from the link with the development of infectious diseases, immune dysfunction in burns is likely to contribute to other secondary pathologies highlighted in the population studies. The excessive inflammatory response seen in the acute phase of burn healing could contribute to gastrointestinal damage, and changes in gut permeability after burns leads to increased risk of infection and endotoxin absorption [54]. Excessive hypermetabolism and immune changes after burn also have been shown to induce insulin resistance long-term, resulting in the heightened risk of diabetes associated with burn injury [34, 55]. Inflammation, stress and hypermetabolism are likely to play a role in cardiac dysfunction after burn. Catecholamines, which are persistently elevated in burn, induce cardiac dysfunction by inducing Ca^2+^ overload in cardiomyocytes and producing damaging oxidation products [56].

#### Cancer

Data from the population studies demonstrated that burn patients have an increased risk of cancer [25]. The immune system plays an important role in cancer prevention, and therefore suppression of the immune system can lead to an increased risk of cancer [57]. Stress/hormone-induced immune suppression impairs the function of NK cells, which are critical to immune surveillance [58]. In addition, reduced activation of cytotoxic T cells reduces the chance of mutant cells being effectively removed following detection. In general, Th2 immunity is thought to enable tumour cells to evade immune surveillance more effectively [59]. Stress hormones also stimulate cell migration and invasion, suggesting a potential direct role in cancer growth and progression. For example, norepinephrine has been shown to increase the invasiveness of nasopharyngeal and ovarian cancer cells via the induction of matrix metalloproteinases which regulates angiogenesis [43]. High levels of histamine and mast cells have also been found in colorectal and breast cancer tissues [60].

#### Cancer risk and gender dimorphism postburn injury

Acute and long-term outcomes for burn patients are impacted by gender. In non-burn trauma, females generally have lower mortality and a lower risk of complications such as sepsis and organ failure as a result of more efficient innate and adaptive immune responses [61]. However, in burn, this is reversed, with males showing a lower risk of secondary complications and having an overall better prognosis [62, 63]. As mentioned previously, female burn patients have a heightened risk of cancer; however, the WA population study found no difference in cancer incidence between male burn patients and uninjured controls [24]. This is a significant finding, especially considering males generally face a higher risk of cancer [64]. It is well known that the immune response is gender dimorphic, and sex hormones are likely to play an important role. Understanding this dimorphism and how it impacts outcomes after burn injury may provide vital clues to the mechanisms underlying the increase in cancer susceptibility in females.

A study in infected ovariectomised female mice found that they had a higher survival rate than control mice, indicating a role for oestrogen in immune function [65]. However, the effect of oestrogen on immune function is complex and not fully understood. Oestrogen receptors are found on numerous immune cells including B and T cells, NK cells, monocytes and macrophages [66]. In pregnancy, immune responses are altered to prevent foetal rejection, a process modulated by sex hormones including oestrogen and progesterone resulting in reduced activity of macrophages, NK cells and Th1 cells and a higher activity of T regulatory cells [67]. This is similar to the immune phenotype seen after burn injury. Pregnant women are also more susceptible to infectious diseases such as influenza [68]. Bird et al. have shown that physiological levels of oestrogen stimulate the immune response, while high levels of oestrogen such as those found in pregnancy have the opposite effect, causing immunosuppression [66]. Burn injury causes an increase in oestrogen levels in mice, and it has been hypothesised that this results in levels resembling pregnancy (immunosuppressive), while levels in male mice reach the levels of uninjured females (immunostimulatory) [69]. These results provide evidence for a likely role of oestrogen in gender dimorphism in burn injury.

Aside from oestrogen, other hormones may also play a role. Prostaglandin E2, which plays a role in mediating the cellular immune response by inhibiting T cell proliferation and macrophage antigen presentation, was shown to be increased in burn-injured female but not male mice 10 days post-injury [70]. Another factor that could play a role in burn injury gender dimorphism is mast cells. Mast cells are regarded as effector cells of allergic reactions, stimulating a Th2-type response. Mackey et al. showed that gene expression in mast cells is significantly different between males and females, with more than 8000 differentially expressed genes [71]. In mice, female mast cells were shown to possess an increased capacity for mediator synthesis and contained higher levels of histamine, tryptase, and chymase in their granules, which are released during times of stress and cause vasodilation, increased vascular permeability and increased production of reactive oxygen species [71]. The increased activation of mast cells in females following burn could contribute to poorer outcomes in both the short and long term.

**Fig. 2:**
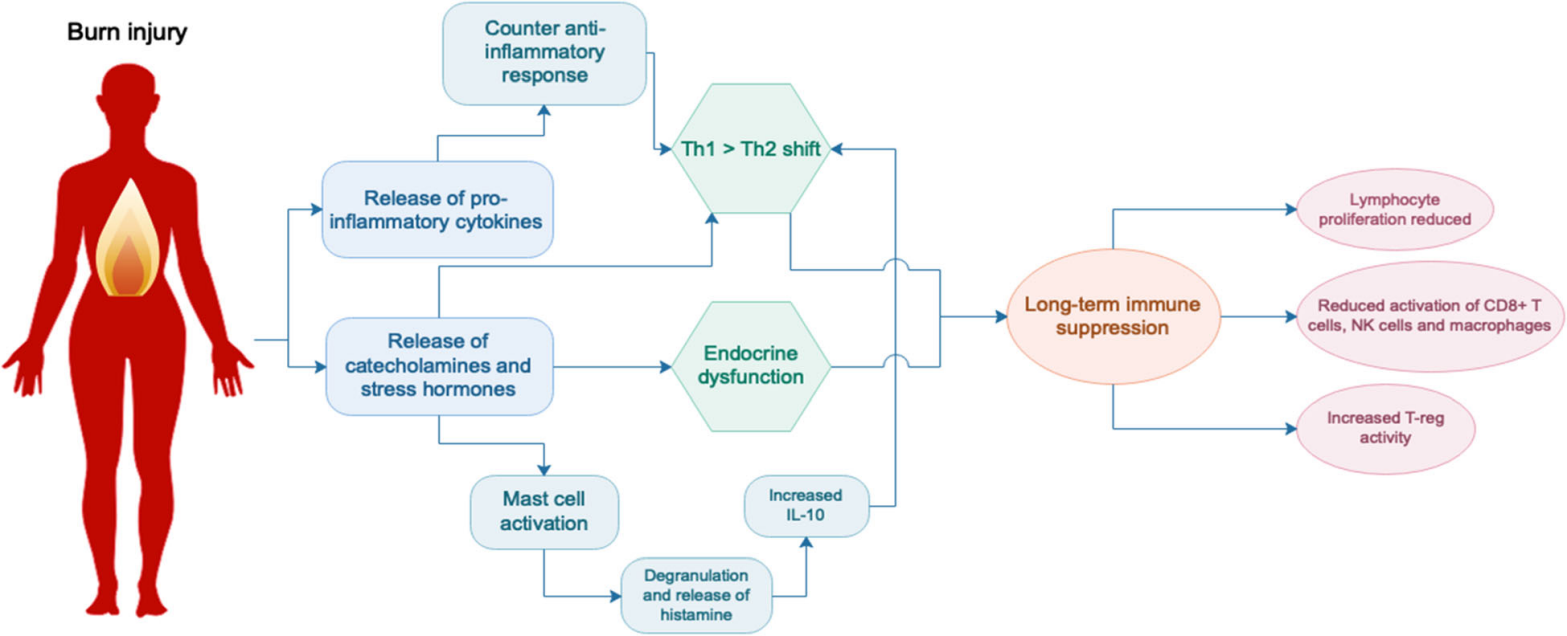
Endocrine and immune system changes following burn injury. Burn injury triggers the immediate release of pro-inflammatory cytokines, catecholamines and stress hormones, followed by a counter anti-inflammatory response and a shift towards a T helper type 2 (Th2) immune environment. Activation of mast cells contributes to this phenotype which is thought to be sustained, resulting in long-term suppression of the immune system. *IL* interleukin, *NK* natural killer

In summary, burn injury is associated with a rapid influx of stress hormones and inflammatory factors resulting in a hyperactive acute innate response, followed by a switch to a Th2-type immune response and subsequent immune suppression that is sustained long-term (Fig. 2). We hypothesise that sustained immune suppression and disruption of homeostasis following burn injury underpins the development of numerous secondary pathologies. Stress arising from the burn and other factors such as pain may exacerbate this immune suppression [16], so better management of burn injury in the clinic could already be improving the impact of burn on immunity. However, more research needs to be done to fully understand the impact of burn on the immune system and the mechanisms that underpin the persistence of immune dysfunction, as well as whether there are specific patient groups at risk. Future studies will then enable the development of preventative treatments that could ideally be administered during the acute healing phase of burn care in order to reduce the risk of secondary complications. This will be beneficial to both the individual and the community by increasing the quality of life of burn survivors and reducing the burden on the health system and families of patients. Considering many burn patients are children and may face these complications relatively early in life, understanding burn injury as a chronic disease is an important step towards better burn care. In addition, the strong links between non-severe burn and secondary complications highlight the need for more in-depth studies on non-severe burns as opposed to severe burns, which to date have received more focus in the research community.

## Conclusions

While acute clinical treatment for burns has improved significantly over the past few decades resulting in significantly higher rates of survival, there is increasing evidence of lifelong impacts of burn injury. Recent findings suggest burn injury can be considered a chronic disease, with secondary morbidity most likely linked to sustained changes to immune function. Future studies to understand the mechanisms involved will be critical to change clinical treatment pathways and reduce the long-term burden of burn injury for patients.

## Data Availability

N/A

## Abbreviations

GH: Growth hormone
GM-CSF: Granulocyte-macrophage colony-stimulating factor
MCP1: Monocyte chemoattractant protein 1
MIP: Macrophage inflammatory protein
NK: Natural killer
NKT: Natural killer T
PTSD: Post-traumatic stress disorder
TBSA: Total body surface area
TNF-α: Tumour necrosis factor alpha
WA: Western Australia

## References

[1] WHO (2018). Burns fact sheet.

[2] Porter C, Tompkins RG, Finnerty CC, Sidossis LS, Suman OE, Herndon DN (2016). The metabolic stress response to burn trauma: current understanding and therapies. Lancet.

[3] Clark A, Imran J, Madni T, Wolf SE (2017). Nutrition and metabolism in burn patients. Burns Trauma.

[4] Jeschke MG, Gauglitz GG, Kulp GA, Finnerty CC, Williams FN, Kraft R (2011). Long-term persistance of the pathophysiologic response to severe burn injury. PLoS One.

[5] Pereira C, Murphy K, Jeschke M, Herndon DN (2005). Post burn muscle wasting and the effects of treatments. Int J Biochem Cell Biol.

[6] Klein GL, Herndon DN, Langman CB, Rutan TC, Young WE, Pembleton G (1995). Long-term reduction in bone mass after severe burn injury in children. J Pediatr.

[7] Chao T, Herndon DN, Porter C, Chondronikola M, Chaidemenou A, Abdelrahman DR (2015). Skeletal muscle protein breakdown remains elevated in pediatric burn survivors up to one-year post-injurY. Shock.

[8] Porter C, Herndon DN, Borsheim E, Bhattarai N, Chao T, Reidy PT (2016). Long-term skeletal muscle mitochondrial dysfunction is associated with hypermetabolism in severely burned children. J Burn Care Res.

[9] St-Pierre DM, Choiniere M, Forget R, Garrel DR (1998). Muscle strength in individuals with healed burns. Arch Phys Med Rehabil.

[10] Klein GL (2015). Disruption of bone and skeletal muscle in severe burns. Bone Res.

[11] Ehrlich HP, Desmouliere A, Diegelmann RF, Cohen IK, Compton CC, Garner WL (1994). Morphological and immunochemical differences between keloid and hypertrophic scar. Am J Pathol.

[12] Finnerty CC, Jeschke MG, Branski LK, Barret JP, Dziewulski P, Herndon DN (2016). Hypertrophic scarring: the greatest unmet challenge after burn injury. Lancet.

[13] Ehde DM, Patterson DR, Wiechman SA, Wilson LG (2000). Post-traumatic stress symptoms and distress 1 year after burn injury. J Burn Care Rehabil.

[14] Ter Smitten MH, de Graaf R, Van Loey NE (2011). Prevalence and co-morbidity of psychiatric disorders 1-4 years after burn. Burns.

[15] Duke JM, Randall SM, Boyd JH, Wood FM, Fear MW, Rea S (2018). A population-based retrospective cohort study to assess the mental health of patients after a non-intentional burn compared with uninjured people. Burns.

[16] Dauber A, Osgood PF, Breslau AJ, Vernon HL, Carr DB (2002). Chronic persistent pain after severe burns: a survey of 358 burn survivors. Pain Med.

[17] Dalal PK, Saha R, Agarwal M (2010). Psychiatric aspects of burn. Indian J Plast Surg.

[18] Duke JM, Rea S, Boyd JH, Randall SM, Wood FM (2015). Mortality after burn injury in children: a 33-year population-based study. Pediatrics.

[19] Duke JM, Boyd JH, Randall SM, Wood FM (2015). Long term mortality in a population-based cohort of adolescents, and young and middle-aged adults with burn injury in Western Australia: A 33-year study. Accid Anal Prev.

[20] Duke JM, Boyd JH, Rea S, Randall SM, Wood FM (2015). Long-term mortality among older adults with burn injury: a population-based study in Australia. Bull World Health Organ.

[21] Mason SA, Nathens AB, Byrne JP, Diong C, Fowler RA, Karanicolas PJ, et al. Increased rate of long-term mortality among burn survivors: a population-based matched cohort study. Ann Surg. 2018. 10.1097/SLA.0000000000002722 31082920

[22] Nitzschke S, Offodile AC, Cauley RP, Frankel JE, Beam A, Elias KM (2017). Long term mortality in critically ill burn survivors. Burns.

[23] Onarheim H, Vindenes HA (2005). High risk for accidental death in previously burn-injured adults. Burns.

[24] Duke J, Rea S, Semmens J, Edgar DW, Wood F (2012). Burn and cancer risk: a state-wide longitudinal analysis. Burns.

[25] Duke JM, Bauer J, Fear MW, Rea S, Wood FM, Boyd J (2014). Burn injury, gender and cancer risk: population-based cohort study using data from Scotland and Western Australia. BMJ Open.

[26] Duke JM, Randall SM, Wood FM, Boyd JH, Fear MW (2017). burns and long-term infectious disease morbidity: a population-based study. Burns.

[27] Fear VS, Boyd JH, Rea S, Wood FM, Duke JM, Fear MW (2017). Burn injury leads to increased long-term susceptibility to respiratory infection in both mouse models and population studies. PLoS One.

[28] Boyd JH, Wood FM, Randall SM, Fear MW, Rea S, Duke JM (2017). Effects of pediatric burns on gastrointestinal diseases: a population-based study. J Burn Care Res.

[29] Stevenson AW, Randall SM, Boyd JH, Wood FM, Fear MW, Duke JM (2017). Burn leads to long-term elevated admissions to hospital for gastrointestinal disease in a West Australian population based study. Burns.

[30] Duke JM, Randall SM, Fear MW, Boyd JH, Rea S, Wood FM (2015). Long-term effects of pediatric burns on the circulatory system. Pediatrics.

[31] Hundeshagen G, Herndon DN, Clayton RP, Wurzer P, McQuitty A, Jennings K (2017). Long-term effect of critical illness after severe paediatric burn injury on cardiac function in adolescent survivors: an observational study. Lancet Child Adolesc Health.

[32] Duke JM, Randall SM, Fear MW, Boyd JH, Rea S, Wood FM (2016). Understanding the long-term impacts of burn on the cardiovascular system. Burns.

[33] O'Halloran E, Shah A, Dembo L, Hool L, Viola H, Grey C (2016). The impact of non-severe burn injury on cardiac function and long-term cardiovascular pathology. Sci Rep.

[34] Duke JM, Randall SM, Fear MW, Boyd JH, O'Halloran E, Rea S (2016). Increased admissions for diabetes mellitus after burn. Burns.

[35] Randall SM, Fear MW, Wood FM, Rea S, Boyd JH, Duke JM (2015). Long-term musculoskeletal morbidity after adult burn injury: a population-based cohort study. BMJ Open.

[36] Duke JM, Randall SM, Fear MW, Boyd JH, Wood FM (2017). Fracture admissions after burns: a retrospective longitudinal study. Burns.

[37] Duke JM, Randall SM, Fear MW, Boyd JH, Rea S, Wood FM (2015). Increased admissions for musculoskeletal diseases after burns sustained during childhood and adolescence. Burns.

[38] Holavanahalli RK, Helm PA, Kowalske KJ (2016). Long-term outcomes in patients surviving large burns: the musculoskeletal system. J Burn Care Res.

[39] Polychronopoulou E, Herndon DN, Porter C (2018). The long-term impact of severe burn trauma on musculoskeletal health. J Burn Care Res.

[40] Vetrichevvel TP, Randall SM, Fear MW, Wood FM, Boyd JH, Duke JM (2016). Burn injury and long-term nervous system morbidity: a population-based cohort study. BMJ Open.

[41] Duke JM, Randall SM, Fear MW, Boyd JH, Rea S, Wood FM. Burn induced nervous system morbidity among burn and non-burn trauma patients compared with non-injured people. Burns. 2019. 10.1016/j.burns.2018.06.006 31056206

[42] Elenkov IJ, Chrousos GP (1999). Stress hormones, Th1/Th2 patterns, pro/anti-inflammatory cytokines and susceptibility to disease. Trends Endocrinol Metab.

[43] Webster Marketon JI, Glaser R (2008). Stress hormones and immune function. Cell Immunol.

[44] Jeschke MG, Mlcak RP, Finnerty CC, Norbury WB, Gauglitz GG, Kulp GA (2007). Burn size determines the inflammatory and hypermetabolic response. Crit Care.

[45] Al-Tarrah K, Moiemen N, Lord JM (2017). The influence of sex steroid hormones on the response to trauma and burn injury. Burns Trauma.

[46] Takagi K, Suzuki F, Barrow RE, Wolf SE, Herndon DN (1998). Recombinant human growth hormone modulates Th1 and Th2 cytokine response in burned mice. Ann Surg.

[47] Mace JE, Park MS, Mora AG, Chung KK, Martini W, White CE (2012). Differential expression of the immunoinflammatory response in trauma patients: burn vs. non-burn. Burns.

[48] O'Sullivan ST, O'Connor TP (1997). Immunosuppression following thermal injury: the pathogenesis of immunodysfunction. Br J Plast Surg.

[49] Hunt JP, Hunter CT, Brownstein MR, Giannopoulos A, Hultman CS, deSerres S (1998). The effector component of the cytotoxic T-lymphocyte response has a biphasic pattern after burn injury. J Surg Res.

[50] MacConmara MP, Maung AA, Fujimi S, McKenna AM, Delisle A, Lapchak PH (2006). Increased CD4+ CD25+ T regulatory cell activity in trauma patients depresses protective Th1 immunity. Ann Surg.

[51] Hanschen M, Tajima G, O'Leary F, Ikeda K, Lederer JA (2011). Injury induces early activation of T-cell receptor signaling pathways in CD4+ regulatory T cells. Shock.

[52] Valvis SM, Waithman J, Wood FM, Fear MW, Fear VS (2015). The immune response to skin trauma is dependent on the etiology of injury in a mouse model of burn and excision. J Invest Dermatol.

[53] Kartchner LB, Gode CJ, Dunn JLM, Glenn LI, Duncan DN, Wolfgang MC (2019). One-hit wonder: late after burn injury, granulocytes can clear one bacterial infection but cannot control a subsequent infection. Burns.

[54] Ziegler TR, Smith RJ, O'Dwyer ST, Demling RH, Wilmore DW (1988). Increased intestinal permeability associated with infection in burn patients. Arch Surg.

[55] Gauglitz GG, Herndon DN, Kulp GA, Meyer WJ, Jeschke MG (2009). Abnormal insulin sensitivity persists up to three years in pediatric patients post-burn. J Clin Endocrinol Metab.

[56] Adameova A, Abdellatif Y, Dhalla NS (2009). Role of the excessive amounts of circulating catecholamines and glucocorticoids in stress-induced heart disease. Can J Physiol Pharmacol.

[57] Finn OJ (2012). Immuno-oncology: understanding the function and dysfunction of the immune system in cancer. Ann Oncol.

[58] Reiche EM, Nunes SO, Morimoto HK (2004). Stress, depression, the immune system, and cancer. Lancet Oncol.

[59] Moreno-Smith M, Lutgendorf SK, Sood AK (2010). Impact of stress on cancer metastasis. Future Oncol.

[60] Elenkov IJ, Webster E, Papanicolaou DA, Fleisher TA, Chrousos GP, Wilder RL (1998). Histamine potently suppresses human IL-12 and stimulates IL-10 production via H2 receptors. J Immunol.

[61] Haider AH, Crompton JG, Oyetunji T, Stevens KA, Efron DT, Kieninger AN (2009). Females have fewer complications and lower mortality following trauma than similarly injured males: a risk adjusted analysis of adults in the National Trauma Data Bank. Surgery.

[62] Karimi K, Faraklas I, Lewis G, Ha D, Walker B, Zhai Y (2017). Increased mortality in women: sex differences in burn outcomes. Burns Trauma.

[63] Wasiak J, Lee SJ, Paul E, Shen A, Tan H, Cleland H (2017). Female patients display poorer burn-specific quality of life 12 months after a burn injury. Injury.

[64] Dorak MT, Karpuzoglu E (2012). Gender differences in cancer susceptibility: an inadequately addressed issue. Front Genet.

[65] Plackett TP, Deburghraeve CR, Palmer JL, Gamelli RL, Kovacs EJ (2016). Effects of estrogen on bacterial clearance and neutrophil response after combined burn injury and wound infection. J Burn Care Res.

[66] Bird MD, Karavitis J, Kovacs EJ (2008). Sex differences and estrogen modulation of the cellular immune response after injury. Cell Immunol.

[67] Robinson DP, Klein SL (2012). Pregnancy and pregnancy-associated hormones alter immune responses and disease pathogenesis. Horm Behav.

[68] Kourtis AP, Read JS, Jamieson DJ (2014). Pregnancy and infection. N Engl J Med.

[69] Gregory MS, Duffner LA, Faunce DE, Kovacs EJ (2000). Estrogen mediates the sex difference in post-burn immunosuppression. J Endocrinol.

[70] Gregory MS, Duffner LA, Hahn EL, Tai HH, Faunce DE, Kovacs EJ (2000). Differential production of prostaglandin E(2) in male and female mice subjected to thermal injury contributes to the gender difference in immune function: possible role for 15-hydroxyprostaglandin dehydrogenase. Cell Immunol.

[71] Mackey E, Ayyadurai S, Pohl CS, D’ Costa S, Li Y, Moeser AJ (2016). Sexual dimorphism in the mast cell transcriptome and the pathophysiological responses to immunological and psychological stress. Biol Sex Differ.

